# Urethral Leiomyoma in Pregnancy: A Rare Case of Symptomatic Pelvic Mass With Successful Surgical Management

**DOI:** 10.7759/cureus.86720

**Published:** 2025-06-25

**Authors:** Rana Beloulou Latoui, Abdul Samed Sulemana, Minhal Chaudhry, Usama Abbas, Muhammad Abdullah Khan

**Affiliations:** 1 Obstetrics and Gynecology, Centre Hospitalo-Universitaire Ibn Rochd Hospital, Annaba, DZA; 2 Renal Medicine, Royal Devon University Hospital NHS Foundation Trust, Exeter, GBR; 3 Obstetrics and Gynaecology, Mampong Government Hospital, Mampong, GHA; 4 Internal Medicine, King Edward Medical University, Lahore, PAK; 5 Physiology, University College of Medicine and Dentistry, University of Lahore, Lahore, PAK; 6 General Medicine, King's Mill Hospital, Nottinghamshire, GBR

**Keywords:** fibroid uterus, leiomyoma, lower urinary tract symptoms, protruding vaginal mass, urethral leiomyoma, uterus like mass

## Abstract

Leiomyomas, benign tumors derived from smooth muscle, are typically found in the uterus. Urethral involvement is an exceptionally rare occurrence, particularly in the context of a pre-fashioned thigh flap. Our case, therefore, presents a unique and intriguing instance, with only a few similar cases reported. We present a case of a 27-year-old primigravida at 11 weeks gestation with a protruding anterior vaginal mass, pelvic heaviness, and recurrent urinary complaints. A well-circumscribed vascular mass came out through the vulval orifice on physical examination. Transvaginal ultrasonography and pelvic magnetic resonance imaging revealed a 6×4 cm-sized mass that originated from the anterior vaginal wall, and it did not invade the adjacent structures. The surgical resection through anterior vaginal route with the use of spinal anesthesia. Histopathological examination revealed urethral leiomyoma, and the diagnosis was supported by positive immunoreactivity for the smooth muscle markers. The recovery following surgery was uneventful, and the pregnancy was carried to term with a successful normal delivery, instilling hope and optimism in similar cases. Our case underscores the crucial role of obstetricians and gynecologists in considering urethral leiomyoma in the differential diagnosis of anterior vaginal wall masses, even in pregnancy. This emphasis on early multimodal treatment can significantly impact the outcome; good results can be achieved, and pregnancy outcomes can be improved with curative therapy.

## Introduction

Leiomyomas, benign mesenchymal smooth muscle cell tumors, are commonly found in the uterus, digestive tract, and skin [1]. However, urethral leiomyomas are rare, representing less than 5% of all paraurethral masses in women [2]. Less than 100 cases have been documented in the literature thus far; these tumors are a unique and intriguing medical phenomenon [2]. The average age of diagnosis is in the fourth decade, and they usually affect women who are of childbearing age [2,3]. However, because they are uncommon during pregnancy, diagnosing and treating them is a difficult and fascinating part of medicine [2,3]. The pathogenesis is not well known, but the finding of estrogen and progesterone receptors in certain lesions makes a hormonal variation, such as that of pregnancy, significant [3]. During pregnancy, these hormones are increased, which can potentially stimulate the growth of urethral leiomyomas and exacerbate their symptoms [3,4]. Patients with urethral leiomyomas commonly manifest with vague lower urinary tract symptoms such as painful micturition, urinary frequency, recurrent urinary tract infections, dyspareunia, or a feeling of a mass [4]. These symptoms may be similar to those of other pelvic pathologies, such as urethral diverticulum, Skene's gland cyst, or pelvic organ prolapse, rendering the diagnosis difficult [5]. Imaging techniques such as transvaginal ultrasound and magnetic resonance imaging (MRI) give practical information about the lesion and its relationship with adjacent structures [5]. Still, histopathological confirmation is the only way to make a definitive diagnosis [4,5]. Surgical excision is the main treatment, especially when the patient is symptomatic or malignancy cannot be excluded [5,6]. This case report emphasizes the rare reproductive age woman with urethral leiomyoma in early pregnancy and suggests a multidisciplinary approach in a similar situation. This case complements the scarce literature on urethral leiomyomas in pregnancy and emphasizes the importance of interdisciplinary support to ensure optimal patient management.

## Case presentation

**Figure 1:**
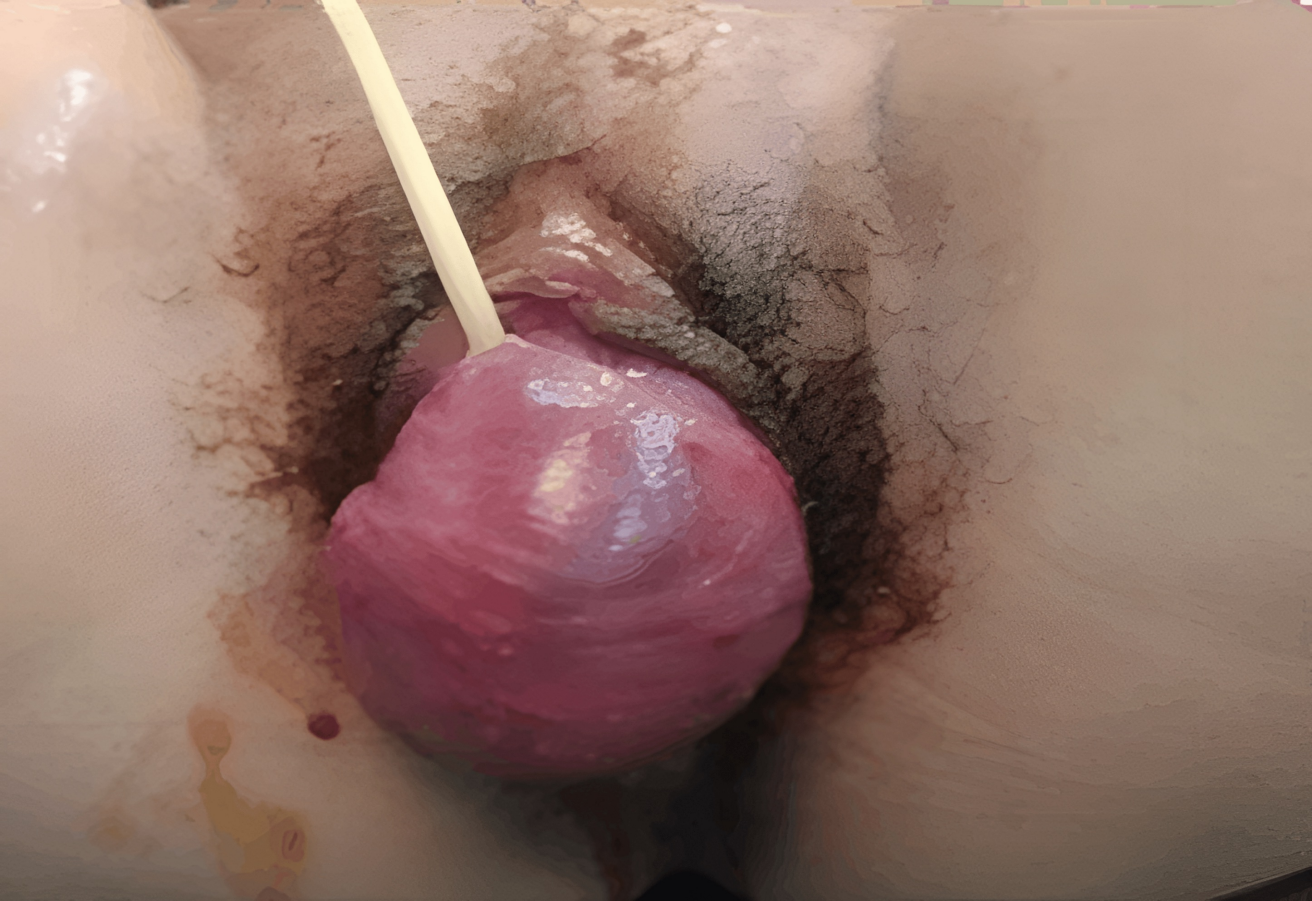
Protrusion of Urethral Leiomyoma Through Anterior Vaginal Mass

A 27-year-old primigravida, married for six months, presented at 11 weeks and 2 days of gestation with a protruding vaginal mass, pelvic heaviness, and urinary discomfort persisting for six months. Notably, she had a history of recurrent urinary tract and vaginal infections, predating the pregnancy. She denied hematuria, fever, or other constitutional symptoms but reported dysuria, mild dyspareunia, and intermittent urinary incontinence due to the vaginal bulge. On physical examination, a firm, circumscribed, nonulcerated mass approximately 10 cm in external dimension was seen protruding through the vulvar introitus. The mass appeared to arise from the anterior vaginal wall, thereby causing anterior displacement and mild dilation of the urethral orifice. The uterus was of appropriate size for gestation, with no adnexal mass palpated. No other abnormalities were noted during the pelvic examination. Figure 1 shows the protrusion of pelvic mass through the anterior vaginal wall.

**Figure 2:**
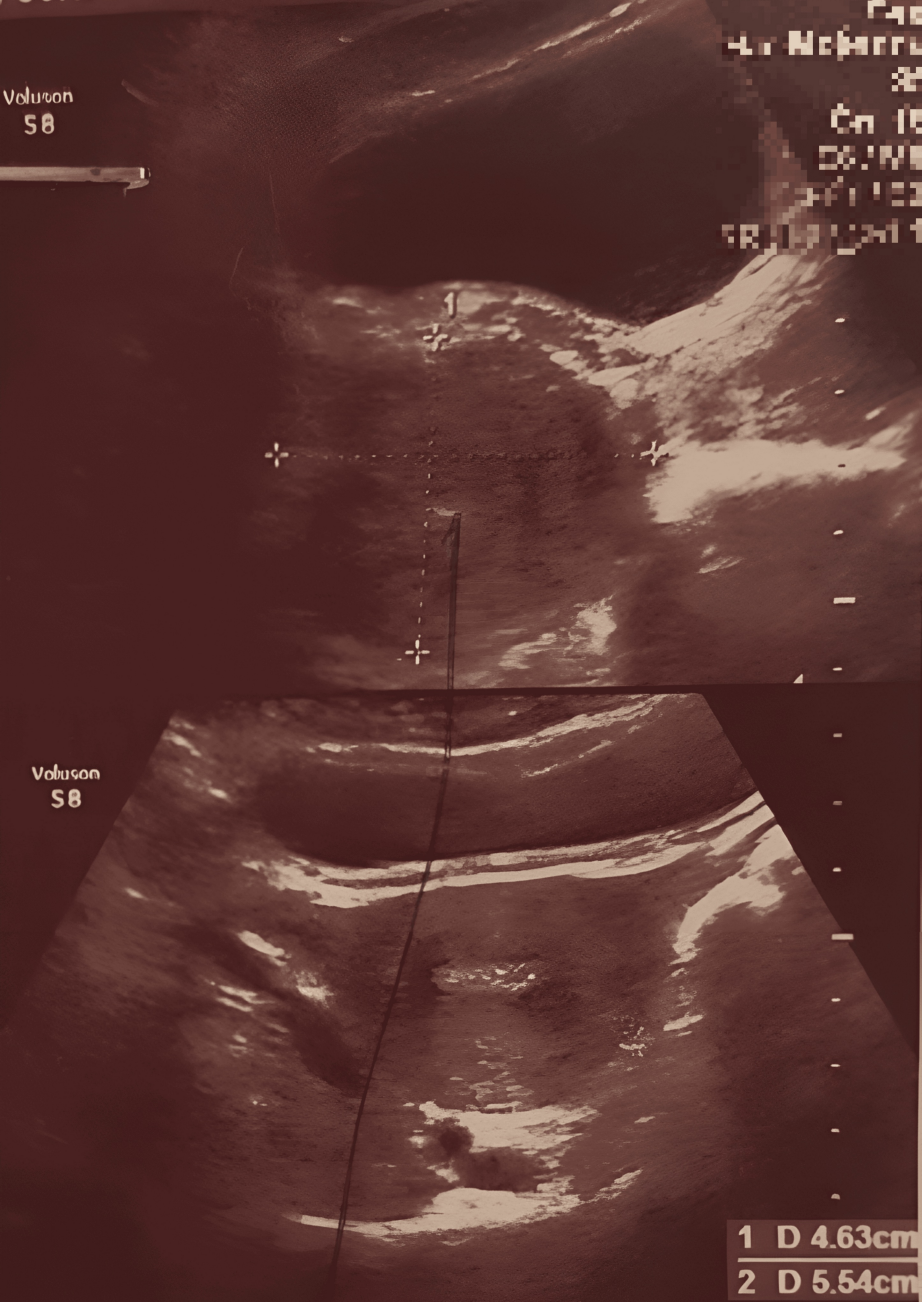
Transvaginal Ultrasound Image Showing a Vascularized Mass Arising From the Anterior Vaginal Wall

Transabdominal and transvaginal ultrasounds were performed. The transabdominal scan confirmed an ongoing intrauterine pregnancy with normal crown-rump length and homogeneous trophoblastic tissue. The transvaginal scan demonstrated a homogeneous, well-vascularized mass measuring 6 × 4 cm, arising from the anterior vaginal wall, separated from the uterus, cervix, and adnexa, and with no obvious infiltration to the adjacent organs, as shown in Figure 2.

**Figure 3:**
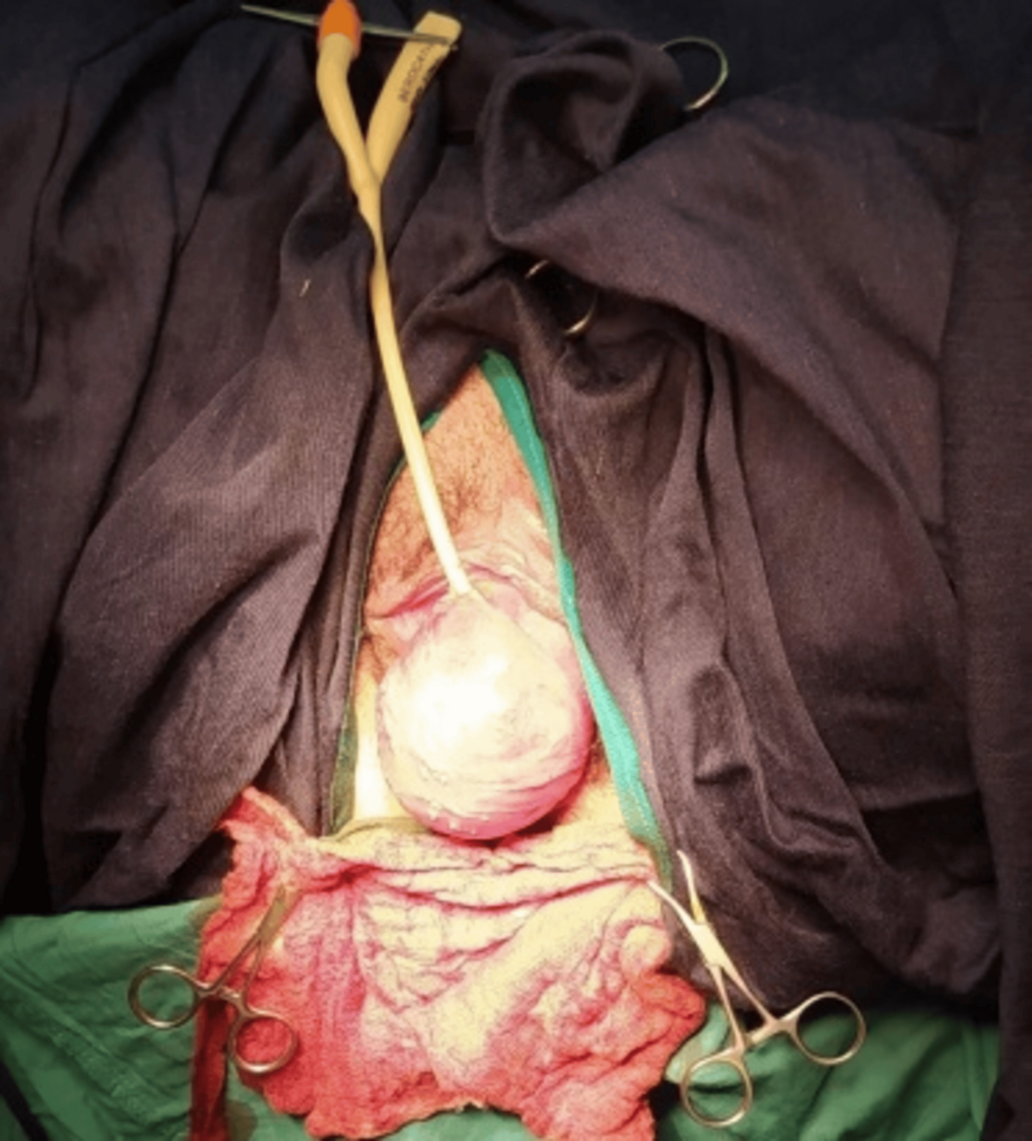
Intraoperative View of Urethral Leiomyoma

MRI additionally characterized the lesion as a well-defined, encapsulated mass compressing the urethra, sparing the bladder and surrounding pelvic musculature. One *Escherichia coli *strain was isolated in urine culture, which was compatible with her history of recurrent urinary tract infections. Given the patient's worsening symptoms and the pregnancy risk, a collaborative effort involving obstetrics, urology, and anesthesiology recommended surgical excision. Figure 3 shows the exposed urethral leiomyoma before excision, which appears as a well-circumscribed, vascularized mass.

**Figure 4:**
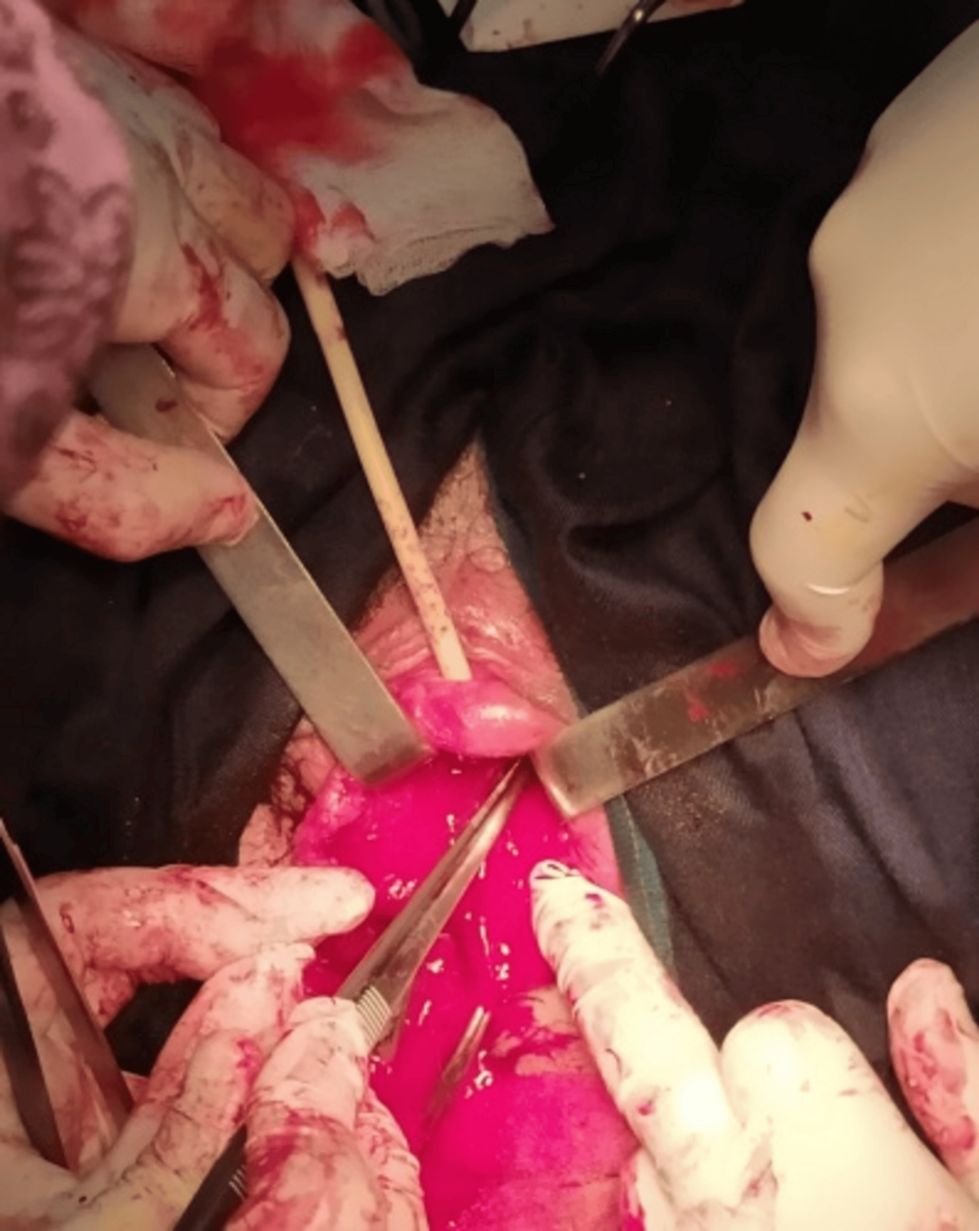
Surgical Approach and Tumor Dissection of Urethral Leiomyoma

Figure 4 illustrates the surgical dissection technique used to separate the leiomyoma from surrounding tissues.

**Figure 5:**
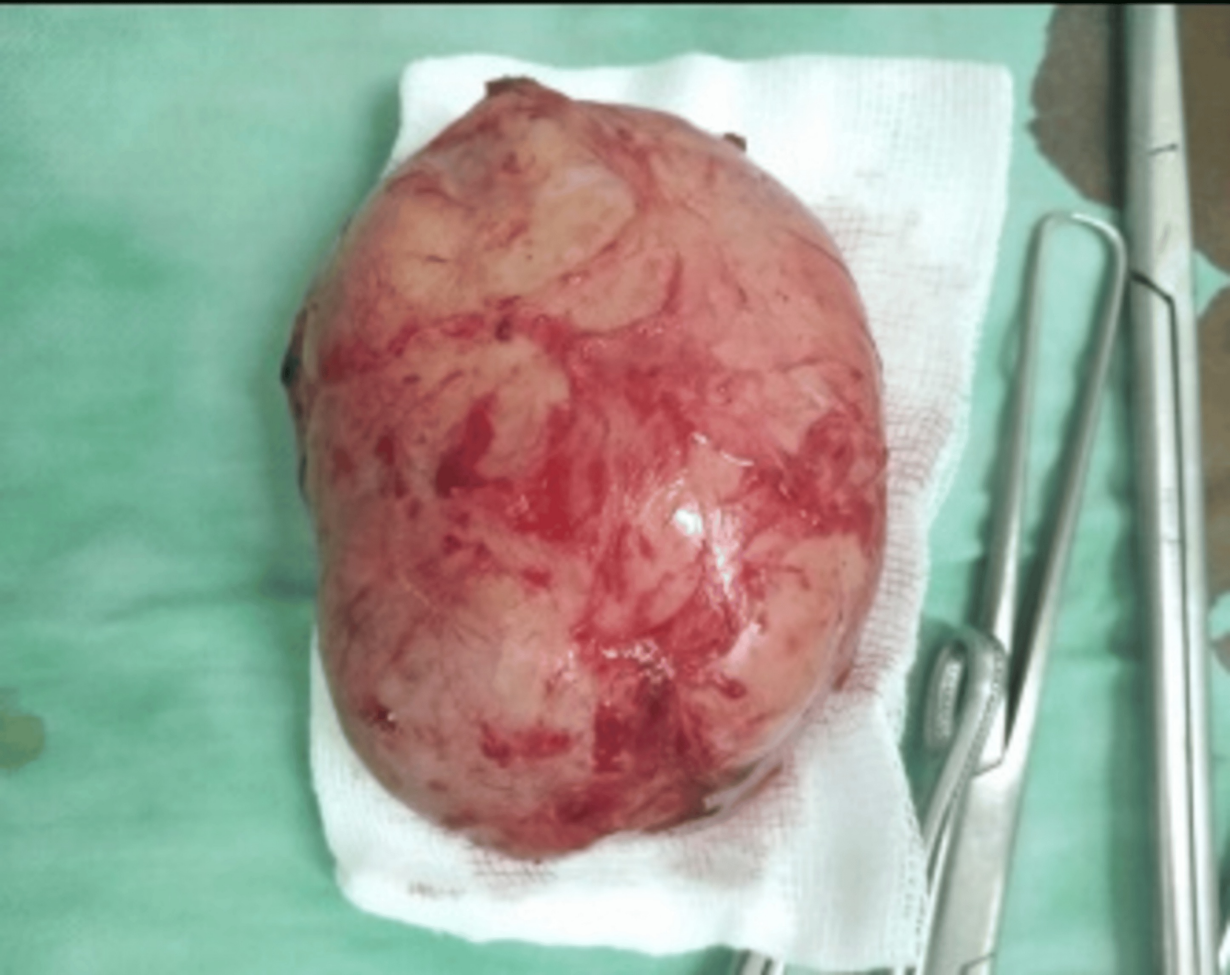
Complete Excision of Urethral Leiomyoma

At 12 weeks gestation, under spinal anesthesia, a vertical anterior vaginal incision was made to access the mass. An encapsulated vascular tumor was carefully dissected from the urethral wall, preserving the sphincteric complex. The cystourethroscopy was done for urethral and bladder integrity was successful without complications. Figure 5 displays the excised tumor, highlighting its size, shape, and encapsulated appearance.

**Figure 6:**
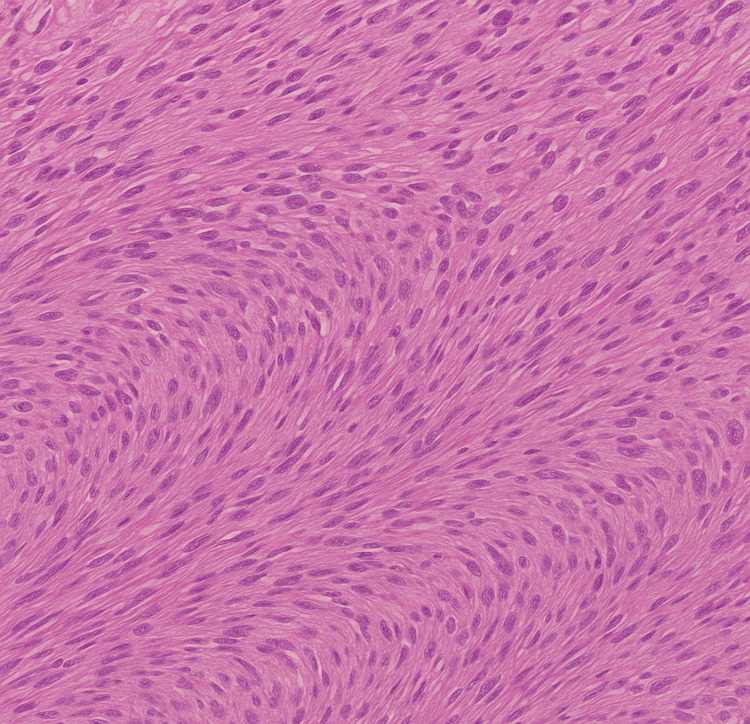
Histopathological Picture of Excised Mass Image is showing bundles of smooth muscle cells.

Histopathological examination showed intersecting bundles of benign smooth muscle cells without cytological atypia or mitotic figures, as shown in Figure 6.

The immunohistochemical stain for desmin and actin confirmed the diagnosis of urethral leiomyoma. Postoperative recovery was uneventful. The patient was asymptomatic afterwards, and no urinary complaints arose. The pregnancy proceeded without complications and culminated with the vaginal delivery of a healthy, term infant. At the six-month follow-up, a clinical examination and pelvic ultrasound demonstrated no evidence of recurrence, instilling confidence in the long-term success of the treatment.

## Discussion

Urethral leiomyomas, a variant of benign mesenchymal tumors, are a fascinating medical anomaly, with fewer than 100 documented cases worldwide [1]. These tumors, which originate from the smooth muscle fibers of the urethral wall, are typically estrogen- and progesterone-sensitive, as evidenced by positive hormone receptor expression in many cases [2,3]. Their growth, a subject of much curiosity, may be influenced by hormonal fluctuations, particularly during pregnancy, when elevated estrogen and progesterone levels can drive tumor enlargement [3]. In our patient, a previously asymptomatic mass became clinically apparent and symptomatic during the first trimester, aligning with this intriguing hormonal hypothesis. The clinical presentation of urethral leiomyomas is often non-specific, including lower urinary tract symptoms such as frequency, dysuria, dyspareunia, and urinary incontinence [4]. In some cases, a visible or palpable anterior vaginal mass may be the first noticeable sign [4]. The broad differential includes urethral diverticula, Skene's gland cysts, periurethral abscesses, and urethral carcinoma [4,5]. A combination of imaging and clinical evaluation is necessary for an accurate diagnosis [5]. Due to its accessibility and capacity to evaluate vascularity and lesion features, ultrasound is usually the first-line modality [5]. MRI's superior soft-tissue delineation enables accurate assessment of tumor margins and surrounding structural involvement, which is critical for surgical planning [6,7].

Histopathological examination confirms the diagnosis, typically showing well-circumscribed interlacing bundles of smooth muscle cells without atypia or mitotic activity [7]. Immunohistochemical positivity for desmin and actin, along with hormone receptors, supports the diagnosis and hormonal responsiveness [8,9]. Complete excision is the primary treatment for symptomatic urethral leiomyomas [10]. In our case, the transvaginal approach provided optimal exposure of the anterior wall while preserving the urethral sphincter. Treatment was performed early in the second trimester and completed using spinal anesthesia to minimize risks to the fetus. Most of the published cases in the literature used general anesthesia. A safe multidisciplinary team comprising obstetricians, urologists, and anesthetists was involved in ensuring patient safety.

Comparative analysis with previous literature supports the uniqueness of our case [1-3]. Cicilet et al. (2016) highlighted the pivotal role of imaging modalities, such as ultrasound and MRI, in diagnosing urethral leiomyoma in non-pregnant women presenting with voiding difficulties [1]. Wu et al. (2024) demonstrated the effectiveness of surgical excision in treating urethral leiomyoma, though their study did not delve into intraoperative strategies or address challenges related to pregnancy [2]. Verma et al. (2014) presented a case of urethral leiomyoma diagnosed through imaging, emphasizing MRI findings, but did not explore the implications of hormonal influences during pregnancy [3].

Our case adds value by integrating surgical technique, anesthetic considerations, and maternal-fetal safety into a comprehensive management strategy for a pregnant patient. The prognosis following excision is excellent, with rare recurrence and no cases of malignant transformation reported to date [10]. Asymptomatic cases may be managed conservatively with close monitoring; however, histopathological confirmation remains essential to exclude malignancy and guide appropriate follow-up. This example emphasizes how crucial it is to rule out hormonally responsive urethral leiomyomas when making a differential diagnosis of anterior vaginal wall masses, particularly in patients who are pregnant. This illustrates favorable results for the mother and the fetus and the necessity of interdisciplinary cooperation, customized anesthetic preparation, and early surgery. 

## Conclusions

Urethral leiomyoma, which is seldom a benign tumor, might cause vague vaginal and urine symptoms, especially during pregnancy. This highlights the significance of having a high index of suspicion to make the right diagnosis with the help of imaging and histological tools. Surgical excision constitutes the definitive treatment, giving very good results with a small risk of recurrence. Timely intervention and good outcomes rely on clinicians' awareness and knowledge regarding this rare condition. Successful management underlines the multidisciplinary approaches in managing urethral leiomyomas, with a significant emphasis on weighing hormonal influences in pregnant patients.

## References

[1] Cicilet S, Joseph T, Furruqh F, Biswas A (2016). Urethral leiomyoma: a rare case of voiding difficulty. BMJ Case Rep.

[2] Wu S, Min Z, Wu L, Zhang M, Wu L (2024). A urethral leiomyoma presenting with dysuria: a rare case report. Medicine (Baltimore).

[3] Verma R, Mehra S, Garga UC, Jain N, Bhardwaj K (2014). Imaging diagnosis of urethral leiomyoma, usual tumour at an unusual location. J Clin Diagn Res.

[4] Sharma R, Mitra SK, Dey RK, Basu S, Das RK (2015). Primary urethral leiomyoma in a female patient: a case report and review of the literature. Afr J Urol.

[5] Ciravolo G, Ferrari F, Zizioli V, Donarini P, Forte S, Sartori E, Odicino F (2019). Laparoscopic management of a large urethral leiomyoma. Int Urogynecol J.

[6] Nozaki S, Naiki T, Hamamoto S (2017). A case of delayed radiation myelopathy of the thoracic vertebrae following low dose radiation therapy for metastatic renal cell carcinoma. Urol Case Rep.

[7] Jiménez Navarro M, Ballesta Martínez B, Rodríguez Talavera J, Amador Robayna A (2019). Recurrence of urethral leiomyoma: a case report. Urol Case Rep.

[8] Alami M, Yousofzai BS, Latoui RB (2024). Prevalence and clinical features of uterine isthmocele following cesarean sections: an observational study at Rabia Balkhi Hospital in Afghanistan. Cureus.

[9] Yousofzai BS, Alami M, Sheela SK (2024). Mifepristone's efficacy for symptomatic relief and size reduction in uterine fibroids: a 2023 prospective observational study at Rabia Balkhi Hospital, Afghanistan. Cureus.

[10] Yousofzai BS, Walizada K, Ahmadi M (2024). Prevalence, clinical features, and management of cervical polyps: a one-year study at Rabia Balkhi Hospital in Afghanistan. Cureus.

